# Simultaneous multi-slice inverse imaging of the human brain

**DOI:** 10.1038/s41598-017-16976-0

**Published:** 2017-12-05

**Authors:** Yi-Cheng Hsu, Ying-Hua Chu, Shang-Yueh Tsai, Wen-Jui Kuo, Chun-Yuan Chang, Fa-Hsuan Lin

**Affiliations:** 1 Institute of Biomedical Engineering, National Taiwan University, Taipei, Taiwan; 2 Institute of Applied Physics, National Chengchi University, Taipei, Taiwan; 3 Research Center for Mind Brain and Learning, National Chengchi University, Taipei, Taiwan; 4 Institute of Neuroscience, National Yang-Ming University, Taipei, Taiwan; 5 Department of Neuroscience and Biomedical Engineering, Aalto University, Espoo, Finland

## Abstract

Ultrafast functional magnetic resonance imaging (fMRI) can measure blood oxygen level dependent (BOLD) signals with high sensitivity and specificity. Here we propose a novel method: simultaneous multi-slice inverse imaging (SMS-InI) — a combination of simultaneous multi-slice excitation, simultaneous echo refocusing (SER), blipped controlled aliasing in parallel imaging echo-planar imaging (EPI), and regularized image reconstruction. Using a 32-channel head coil array on a 3 T scanner, SMS-InI achieves nominal isotropic 5-mm spatial resolution and 10 Hz sampling rate at the whole-brain level. Compared with traditional inverse imaging, we found that SMS-InI has higher spatial resolution with lower signal leakage and higher time-domain signal-to-noise ratio with the optimized regularization parameter in the reconstruction. SMS-InI achieved higher effective resolution and higher detection power in detecting visual cortex activity than InI. SMS-InI also detected subcortical fMRI signals with the similar sensitivity and localization accuracy like EPI. The spatiotemporal resolution of SMS-InI was used to reveal that presenting visual stimuli with 0.2 s latency between left and right visual hemifield led to 0.2 s relative hemodynamic response latency between the left and right visual cortices. Together, these results indicate that SMS-InI is a useful tool in measuring cortical and subcortical hemodynamic responses with high spatiotemporal resolution.

## Introduction

There is accumulating evidence suggesting the advantage of collecting BOLD signals with sub-second sampling rate. First, we can monitor and suppress cardiac- and respiratory-related physiological noise^1^ more efficiently^2–5^. Second, the fine timing information related to neuronal activity in the range of a few hundreds of milliseconds can be detected in hemodynamic response^6,7^. Third, the functional connectivity in the resting-state networks involving visual and motor cortices is more stably detected in both spatial and temporal domains when the sampling frequency is higher than 0.1 Hz^8^. Fourth, with improved sampling rate, we can measure the effective connectivity between the visual and motor system more reliably, demonstrating the advantage of characterizing inter-regional information flow in simple visuo-motor tasks^9,10^. Lastly, with the high sampling rate, the time series can be cleaned using large number of regressors and achieve higher statistical power in resting state network estimation^11,12^.

There are currently different methods to achieve fMRI measurements with a high sampling rate. Parallel detection methods can achieve 10 Hz sampling rate with millimeter spatial resolution and whole-brain coverage. These include, for example, MR-encephalography (MREG)^13^ and inverse imaging (InI)^14,15^. Targeting at a more homogeneous spatial resolution, MREG uses non-Cartesian^16^ trajectories at the cost of more complicated image reconstruction, where volumetric image voxels are coupled in a large linear system. On the other hand, InI uses a simpler rectilinear *k*-space trajectory to allow the 3D image reconstruction separated into multiple linear systems of a much smaller size. However, InI has rather anisotropic resolution because the sensitivity information from the coil array is not sufficient to separate spatially aliased image voxels^14^.

Recently, parallel-accelerated simultaneous multi-slice (SMS) imaging^17^ using blipped controlled aliasing (CAIPI) EPI has been introduced^18^. This method was further integrated with simultaneous echo refocusing (SER) to achieve a 2.5 Hz volumetric sampling rate in fMRI experiments^7,19^. Using a 32-channel head coil array on 3 T, blipped-CAIPI-EPI provides acquisitions with about 2–3 mm isotropic spatial resolution at a volumetric sampling interval in the range of 300 ms to 600 ms. However, the quality of fMRI time series was found poor if the sampling interval was further reduced to 100 ms^7^. Blipped CAIPI-EPI using regularized SENSE reconstruction has been proposed^20^. Yet its acquisition rate was below 1.7 Hz (TR ranged between 0.6 s and 6 s). Theoretically, the regularization parameter should modulate the *tSNR* and the spatial smearing of the reconstructed images^21^. Lastly, while tuning acquisition parameters of blipped-CAIPI-EPI is expected to achieve TR as short as 100 ms, to the best of our knowledge, no fMRI experiment has actually demonstrated the usefulness of such fast fMRI with the blipped-CAIPI-EPI.

In this study, we proposed an optimized simultaneous multi-slice inverse imaging (SMS-InI), which integrates the state-of-the-art blipped-CAIPI-EPI and SER in image acquisition and regularized reconstruction in order to achieve high spatiotemporal resolution with high computational efficiency. With the optimized volume prescription, slice arrangement, and choice of regularization, we were able to achieve isotropic 5-mm spatial resolution, 100-ms volumetric sampling interval, and high *tSNR* at 3 T MRI using a 32-channel head coil array. Using two-shot blipped-CAIPI-EPI and SER in acquisition and regularized SENSE reconstruction, SMS-InI had much improved *tSNR* (~57). We explicitly investigated how to trade-off between *tSNR* and signal leakage by tuning the regularization parameter in blipped-CAIPI-EPI reconstruction. Functional MRI data reconstructed with slice-GRAPPA and regularized SENSE were also compared. The performance of SMS-InI was empirically quantified and further compared with the traditional InI and EPI in the human visual and sensorimotor systems.

## Materials and Methods

### SMS-InI pulse sequence and imaging parameters

All imaging experiments were performed on a 3 T MRI scanner (Skyra, Siemens, Erlangen, Germany) with a 32-channel head coil array. The pulse sequence of SMS-InI is the combination of SMS imaging with blipped CAIPI^18^ and simultaneous echo refocusing (SER)^19^. To achieve the goal of measuring fMRI with 10 Hz sampling rate, whole-brain coverage, and 5-mm isotropic resolution, we prescribed 20 axial 4-mm thick slices with 1-mm gap between slices. To describe imaging slices, we used 4*n* + 1, 4*n* + 2, 4*n* + 3, and 4*n* + 4 (*n* = 0, …, 4) to indicate the 20 consecutive slices. These slices were separated into two slice groups, each of which had 10 slices and excited/read in 50 ms. Slices 4*n* + 1 and 4*n* + 3 were denoted as slice group 1, while Slices 4*n* + 2 and 4*n* + 4 were denoted as slice group 2. In slice group 1, slices 4*n* + 1 and 4*n* + 3 were separated by SER. Specifically, a dephasing gradient moment in the readout direction (left-right) was applied after the excitation of slices 4*n* + 1 and before the excitation of slices 4*n* + 3. Then, frequency encoded NMR signals were read separately for slices 4*n* + 1 and 4*n* + 3 using oscillating EPI readouts. Note that in SER, the order of the reading slices 4*n* + 1 and 4*n* + 3 alternated between phase encoding lines. The echo time for slices 4*n* + 1 and 4*n* + 3 differed by 2.5 ms. Similarly, slices 4*n* + 2 and 4*n* + 4 in slice group 2 were separated by SER.

While separated by SER, for example, slices 4*n* + 1 from slices 4*n* + 3, all slices indexed by 4*n* + 1 were still aliased, since they were simultaneously excited and read. To further separate these aliased slices, we used blipped CAIPI to move neighboring slices indexed by, for example, 4*n* + 1, by 1/3 FOV in the phase-encoding (anterior-posterior) direction. This allowed us to separate aliased slices more effectively using coil sensitivity information. Other imaging parameters were: flip angle = 30°, in-plane resolution = 5 mm × 5 mm, FOV = 210 × 210 × 100 mm^3^, TR = 100 ms, and TE = 25/27.5 ms for two slice sets in SER. Note that this acceleration factor (R = 5) was chosen after carefully trading-off between the coil sensitivity information (32-channel head coil array at 3 T) and the desired spatiotemporal resolution (10 Hz sampling rate with the whole-brain coverage and the highest possible spatial resolution). Moreover, with double acceleration factor (R = 10), the whole brain image can be acquired with single EPI echo train, single-shot SMS-InI (SMS-InI-sh), and achieve even higher sampling rate.

**Figure 1:**
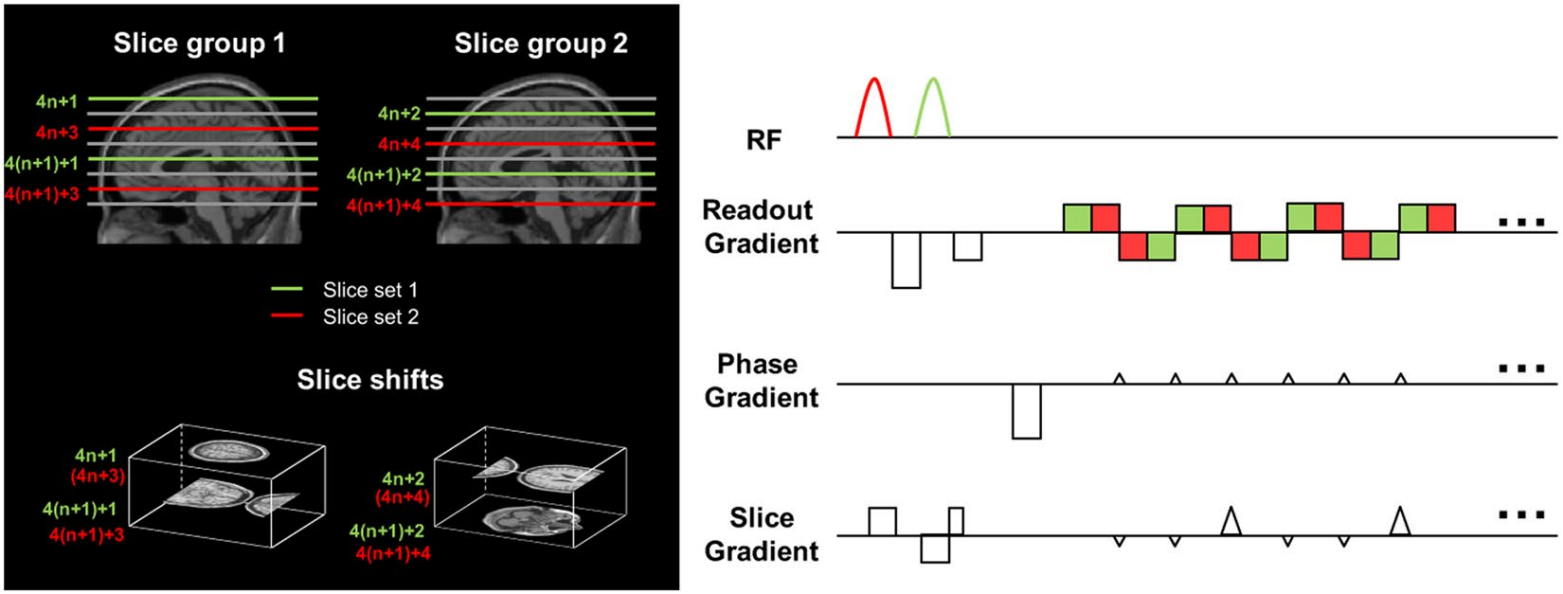
Slice prescription (left) and pulse sequence diagram (right) of SMS-InI. The acquisition used two slice groups, each of which had two slice sets. Two different slice sets were separated by simultaneous echo refocusing (SER). Adjacent slices within the same slice set were shifted by blipped-CAIPI EPI with 1/3 FOV shift. These shifted slices were then separated by coil sensitivity information. The acquisition time for each slice group was 50 ms.

The slice prescription and the pulse sequence diagram of our acquisition are illustrated in Fig. 1.

### SMS-InI image reconstruction

In SMS-InI acquisition, five distinct image pixels were first spatially weighted by coil sensitivity before spatially shifted (along the phase encoding direction) and aliased (along the slice direction) together. The relationship between the observed aliased image pixel and the five un-aliased image pixels to be reconstructed can be described by a linear equation. Instantaneous volumetric images were reconstructed by the regularized SENSE algorithm^21,22^ using simultaneous measurements of aliased image pixels from multiple channels of a coil array:

1 $${\boldsymbol{x}}={({{\bf{A}}}^{H}{{\bf{C}}}^{-1}{\bf{A}}+{\rm{\lambda }}{\bf{I}})}^{-1}{{\bf{A}}}^{H}{{\bf{C}}}^{-1}{\boldsymbol{y}},$$

where ***x*** denotes the vector of five un-aliased image pixels to be reconstructed, ***y*** denotes the vector of observed aliased image pixel from all channels of a coil array, **A** is the image encoding matrix, **C** is the noise covariance matrix, and λ is a regularization parameter. Image pixels reconstructed by Eq. 1 were the analytical solutions of the optimization problem of the regularized SENSE reconstruction:

2 $${\boldsymbol{x}}={{\rm{argmin}}}_{{\bf{x}}^{\prime} }(\parallel {{\bf{C}}}^{-1/2}({\bf{y}}-{\bf{A}}{\bf{x}}^{\prime} ){\parallel }^{2}+{\rm{\lambda }}\parallel {\bf{x}}^{\prime} {\parallel }^{2}).$$

We systematically varied λ that λ/λ_1_ = 10^−4^, 10^−3^, 10^−2^, 10^−1^, 1, 10^1^, 10^2^, 10^3^, 10^4^, where λ_1_ was the largest eigenvalue of **A** ^*H*^ **C** ^−1^ **A**.

The regularization parameter was empirically determined by trading off between the signal leakage (SL) and *tSNR* such that the *tSNR* was high and the SL was minor. Details about the calculation of SL and *tSNR* were described below.

For comparison, we also reconstructed images using the slice-GRAPPA algorithm^18^ and split slice-GRAPPA^23^ algorithm with signal leakage suppression.

### Image encoding matrix

We used the *in vivo* sensitivity method^24^, which takes the empirically measured fully gradient-encoded reference scan from each channel of a coil array as the coil sensitivity maps, to construct the imaging encoding matrix **A**. The *in vivo* sensitivity method is particularly useful for reconstructing fMRI data, because fMRI primarily concerns the relative changes of the BOLD signal over time at each image pixel, rather than the relative contrast between image pixels. Using the *in vivo* sensitivity method also alleviated the challenge of estimating complex-valued coil sensitivity maps compatible to the SMS-InI measurements.

**Figure 2:**
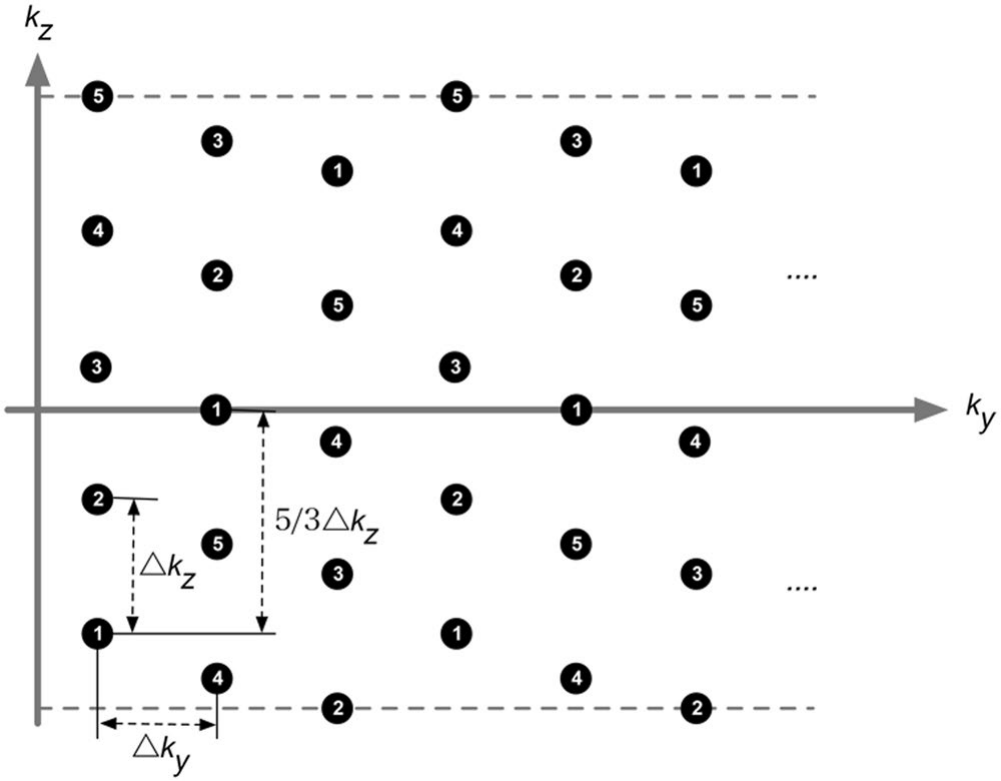
The *k*-space sampling pattern of the reference scan on the *k*_*y*_ *-k*_*z*_ plane with phase encoding direction in the *y* axis and slice direction in the *z* axis with R = 5 with 1/3 FOV shift. The numbers marked on the *k*-space points indicated the order of the partition encoding steps. For example, *k*-space points marked with “1” were sampled during the first partition encoding step. We circularly shifted the order of partition encoding step, such that all sampled *k*-space points were within the half of the inverse of the distance between adjacent simultaneously excited slices. The *k*-space points marked with “1” corresponded to the *k*-space data points in the SMS-InI acquisition.

Note that the SMS-InI acquisition is equivalent to the under-sampled 3D blipped CAIPI-EPI acquisition associated with a zig-zag *k*-space sampling pattern on the *k*_*y*_ *-k*_*z*_ plane with phase encoding direction in the *y* axis and slice direction in the *z* axis^25^. Therefore, the fully gradient encoded reference scan was implemented by adding partition encoding steps to acquire the full *k*-space data on the *k*_*y*_ *-k*_*z*_ plane. Volumetric images can be reconstructed from these reference scan data after the fast Fourier transform. Figure 2 shows the *k*-space sampling pattern of the reference scan for R = 5 with 1/3 FOV shift, where Δk_z_ and Δk_y_ were the inverse of the slice FOV and phase FOV, respectively. The numbers marked on the *k*-space points indicated the order of the partition encoding steps. For example, *k*-space points marked with “1” were sampled during the first partition encoding step. We circularly shifted the order of partition encoding step, such that all sampled *k*-space points were within the half of the inverse of the distance between adjacent simultaneously excited slices. The *k*-space points marked with “1” corresponded to the *k*-space data points in the SMS-InI acquisition. The reconstructed volumetric images of the reference scan using this sampling pattern had the same 1/3 FOV shift as SMS-InI.

### fMRI experiment paradigm and data analysis

Six subjects participated in the study. Prior to the experiment, all subjects gave written informed consents approved by the Institutional Review Board at National Taiwan University Hospital. Two experiments were run in accordance with the guidelines of the Helsinki Declaration. We performed two experiments to study the spatial resolution (Experiment 1) and temporal resolution of (Experiment 2) of SMS-InI. Both experiments were approved by the Institutional Review Board at National Taiwan University Hospital. In Experiment 1, stimuli with full-field checkerboard reversal (8 Hz and 0.5 s duration) were randomly presented to the subjects in a rapid event-related design. The stimuli were presented in either a large (15.7°) or a small (8.1°) visual angle. The rationale for such stimulus design was to test if SMS-InI has the spatial resolution to reveal different sizes of the visual cortex elicited by different stimulus sizes. There were 30 large stimuli and 30 small stimuli in a 4-minute run. A small black crosshair randomly changed its color to red for 0.25 s. Subjects were instructed to press a button when the red crosshair showed up. This crosshair was used to minimize eye movements. Two runs of data were collected for each subject. The stimuli were presented using PsychoPy (version 1.8; http://www.psychopy.org/about/index.html)^26^.

In order to test temporal resolution, in Experiment 2, we randomly presented two hemi-field visual checkerboard patterns separated by 0.2 s (right hemi-field stimulus followed by left hemi-field stimulus; 15.7° view angle; each hemifield stimulus 0.125 s duration) to the subjects. Using this design, we studied if the relative BOLD signal latency between left and right visual cortices represented the relative latency between left and right visual hemi-field stimuli. There were 50 stimuli (right-left hemi-field checkerboard patterns) in a 4-minute run. Like in Experiment 1, a random color-changing crosshair was presented to the subjects and the subjects were instructed to press a button upon seeing the color change. Two runs of data were collected for each subject. The onsets of events in both Experiment 1 and Experiment 2 were randomized with a minimal inter-stimulus interval of 2 s.

All data will be made available upon requests to the corresponding author of this paper.

### EPI, InI, and anatomical MRI acquisitions and reconstruction

EPI and InI data were collected for Experiment 1. Parameters for EPI were: TR = 2 s; TE = 30 ms, flip angle = 90°; FOV = 224 × 224 mm^2^; image matrix = 64 × 64; slice thickness = 3.5 mm without any gap; 33 slices. Two 4-min runs were collected for each subject. Parameters for InI were: TR = 0.1 s; TE = 30 ms; flip angle = 30°; FOV = 256 × 256 × 256 mm^3^; image matrix = 64 × 64 × 64; acceleration (partition-encoding) direction: anterior-posterior. InI was reconstructed by the minimum-norm estimates^15^.

Structural images for each subject were acquired using a 3D *T*_1_-weighted pulse sequence (MP-RAGE: TR/TE/TI = 2,530/3.49/1100 ms, flip angle = 7°, image matrix = 256 × 256 × 192, FOV = 25.6 × 25.6 × 19.2 cm^3^).

### EPI, InI, and SMS-InI processing and ROI definition

EPI data were first pre-processed with SPM (http://www.fil.ion.ucl.ac.uk/spm/software/spm12/; version SPM 12) by applying motion correction, slice-timing correction, normalization, and spatial smoothing (3D isotropic Gaussian kernel with the full-width-half-maximum = 8 mm). InI data were only normalized and spatially smoothed by SPM. SMS-InI was pre-processed like EPI, except that no motion correction was applied. All data were further detrended and high-pass filtered (cut-off frequency = 1/128 Hz).

To estimate BOLD responses in SMS-InI measurements, we used General Linear Model (GLM). In Experiment 1, we used a canonical hemodynamic response function (HRF) to model the BOLD signals elicited by visual stimuli. Specifically, the convolution between onsets of visual stimuli and canonical HRF, together with a constant term, were incorporated into the design matrix of GLM. For EPI data, we also analyzed data with GLM. EPI *t* statistics larger than 6.5 (20484 voxels at cortex; Bonferroni corrected *p* < 0.001) at the group level were taken as the active voxels. For InI and SMS-InI, we used a more stringent threshold (*t* statistics > 15; Bonferroni corrected *p* < 0.0001) because they had 20-fold more data samples than EPI.

In Experiment 2, the region-of-interest for the left and right visual cortex were determined by i) voxels with *t* statistics >15, and ii) excluding voxels no farther away from the central fissure by 1.5 cm in order to minimize potential spatial smearing between left and right visual cortices in the reconstruction and spatial normalization. Then we used GLM with finite impulse response basis functions (300 time points, 6-s pre-stimulus baseline and 24-s post-stimulus duration in 0.1 s steps) to analyze the data in the left and right visual cortical ROIs without *a priori* assumption on the shape of the hemodynamic response.

### Performance quantification

#### fMRI time series quality assessed by time-domain SNR

We measured the *tSNR* as a metric for the quality of fMRI time series. Specifically, given the time series of an image voxel, its *tSNR* was calculated as the ratio between the average of the time series and the standard deviation of the time series.

Note that the chosen regularization parameter can also affect *tSNR*: Eq. 1 shows that a large regularization parameter λ suppresses dynamic changes in a time series and causes a small *tSNR* as the consequence of minimizing the numerator in the *tSNR* calculation. On the other hand, a small regularization parameter cannot suppress the fluctuation caused by the noise amplification in image reconstruction. This also leads to a small *tSNR* because of a large denominator in the *tSNR* calculation. The optimal regularization parameter should provide the highest *tSNR*. We calculated the *tSNR* from images reconstructed by different regularization parameters.

#### Spatial smearing quantified by signal leakage

In simulations, we reconstructed SMS-InI images after seeding a true source signal at a specific location. This procedure allowed us calculating the signal leakage (SL) to evaluate the spatial smearing of SMS-InI reconstruction.

3 $$SL(\mathop{r}\limits^{ \rightharpoonup })=\frac{{\sum }_{{\mathop{{\boldsymbol{r}}}\limits^{{\boldsymbol{ \rightharpoonup }}}}^{\text{'}}\ne \mathop{{\boldsymbol{r}}}\limits^{{\boldsymbol{ \rightharpoonup }}}}{\boldsymbol{x}}(\,\mathop{{\boldsymbol{r}}}\limits^{{\boldsymbol{ \rightharpoonup }}},{\mathop{{\boldsymbol{r}}}\limits^{{\boldsymbol{ \rightharpoonup }}}}^{\text{'}})}{{\sum }_{{\mathop{{\boldsymbol{r}}}\limits^{{\boldsymbol{ \rightharpoonup }}}}^{\text{'}}}{\boldsymbol{x}}(\,\mathop{{\boldsymbol{r}}}\limits^{{\boldsymbol{ \rightharpoonup }}}\,,{\mathop{{\boldsymbol{r}}}\limits^{{\boldsymbol{ \rightharpoonup }}}}^{\text{'}})}\times 100 \% ,$$

where $x(\mathop{r}\limits^{ \rightharpoonup },{\mathop{{\boldsymbol{r}}}\limits^{{\boldsymbol{ \rightharpoonup }}}}^{{\boldsymbol{\text{'}}}})$ represents the SMS-InI reconstructed signal at location $\,{\mathop{{\boldsymbol{r}}}\limits^{{\boldsymbol{ \rightharpoonup }}}}^{{\boldsymbol{\text{'}}}}$ with true source signal at location $\mathop{r}\limits^{\rightharpoonup }$. $SL(\mathop{r}\limits^{ \rightharpoonup })=0$ represents the ideal reconstruction. $SL(\mathop{r}\limits^{ \rightharpoonup })=100 \%$ represents the worst case reconstruction, where reconstructed signals were all shifted away from the source location.

SL can be modulated by the chosen regularization parameter in image reconstruction: a large regularization parameter causes more serious spatial smearing (large SL). This is the consequence of emphasizing the minimization of the image reconstruction prior term, which is the sum of squares of reconstructed image pixels values (${\rm{\lambda }}\parallel {\bf{x}}^{\prime} {\parallel }^{2}$ in Eq. 2), via using a large regularization parameter. Such emphasis can cause more spreading of the image voxel values and consequently larger SL.

We calculated the SL from images reconstructed regularized SENSE method with different choices of the regularization parameters. SL was also calculated from images reconstructed with the slice-GRAPPA method reported in the original blipped-CAIPI-EPI^18^.

#### Sensitivity and specificity of detecting active brain areas using receiver-operating characteristic curves

We evaluated the sensitivity and specificity of InI and SMS-InI reconstructed by slice-GAPPA or regularized SENSE methods with different regularization parameters using Receiver Operating Characteristic (ROC) analysis. This was used to assess the detection power of functional activity measured by InI and SMS-InI. Specifically, the gold standard of the visual cortex was first estimated from EPI data (see sub-section EPI, InI, and SMS-InI processing and ROI definition above). Then we varied the threshold of the *t* statistics derived from InI and SMS-InI data to calculate true-positive rate (TPR) and false-positive rate (FPR). Here the TPR and FPR with α-level significance were calculated as

4 $$TPR(\alpha )=\frac{{\boldsymbol{size}}({\boldsymbol{area}}({\boldsymbol{R}}. > {\boldsymbol{\alpha }})\cap {\boldsymbol{area}}({{\boldsymbol{R}}}_{{\boldsymbol{EPI}}}))}{{\boldsymbol{area}}({{\boldsymbol{R}}}_{{\boldsymbol{EPI}}})}$$

5 $$FPR(\alpha )=\frac{{\boldsymbol{size}}({\boldsymbol{area}}({{\boldsymbol{R}}}_{.} > {\boldsymbol{\alpha }})\cap ({\boldsymbol{area}}({\boldsymbol{Vtx}})-{\boldsymbol{area}}({{\boldsymbol{R}}}_{{\boldsymbol{EPI}}})))}{({\boldsymbol{area}}({\boldsymbol{Vtx}})-{\boldsymbol{area}}({{\boldsymbol{R}}}_{{\boldsymbol{EPI}}}))}$$

Here $area({R}_{EPI})$ and $area(Vtx)$ indicate the visual cortex estimated by EPI data and defined by SPM. $area({R}_{.} > \alpha )$ indicates the areas wi*t*h *t* statistics larger than *α* from either InI or SMS-InI data. ∩ and - denote the Boolean AND and set difference operator, respectively. The operator $size(\cdot )$ calculates the size of the volume. ROC curves for both InI and SMS-InI were calculated separately. The areas under each ROC curve were used to quantify the detection power.

#### Relative latency in visual cortex hemodynamics estimated by cross-correlation

In Experiment 2, the hemodynamic responses at left and right visual cortex were temporally shifted forward and backward relative to each other by + 1 s or −1 s in 0.1 s steps. We calculated the correlation coefficients for each temporally shifted visual cortex time series pairs. The temporal shift corresponding to the largest correlation coefficient was taken as the relative latency between ROI’s.

All image reconstructions and performance quantification were carried out in MATLAB (Mathworks; Natick, MA, USA) on a Linux workstation using Xeon (Intel Santa Clara, CA, USA) Xeon CPU (2.4 GHz) with 64 GBytes of memory.

## Results

### Volumetric images

**Figure 3:**
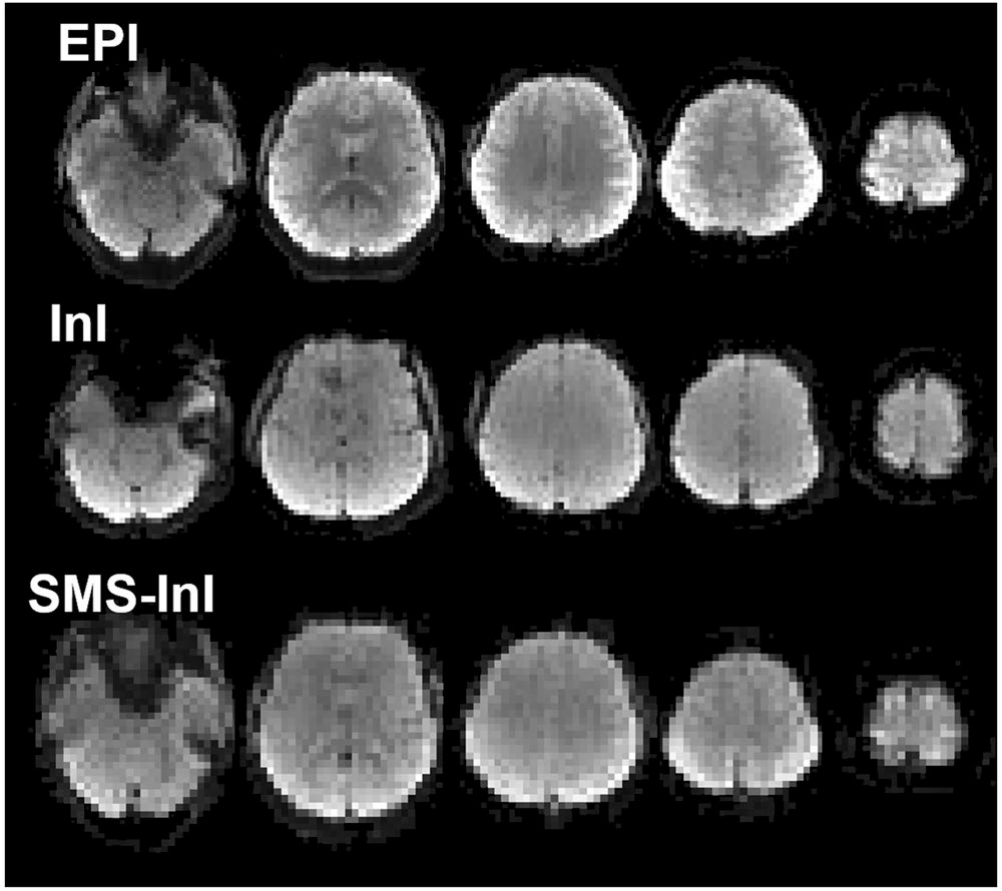
Eight axial slice images from EPI, InI, and SMS-InI. Both EPI and SMS-InI show similar coarse structure feature, while InI is more spatially smooth.

The SMS-InI reconstruction consisted of many independent sets of aliased image voxels related by an encoding matrix of small dimension (32 × 5 in this study). Because of this separation and the analytical solution (Eq. 1), reconstructing a volume of SMS-InI took less than 0.1 seconds. Eight axial slices of EPI, InI, and SMS-InI images from a representative subject were shown in Fig. 3. The spatial and temporal resolution of these method were summarized in Table 1. Structural features in EPI and SMS-InI images were visually similar, while InI was spatially smoothed due to reduced spatial information from the coil array to separate aliased images during the acquisition.

**Table 1:** The nominal resolution and temporal resolution of EPI, InI, and SMS-InI methods used in this study.

Pulse sequence	Nominal resolution	Temporal resolution
EPI	3 mm × 3 mm × 3 mm	2 s
InI	4 mm × 4 mm × 4 mm	0.1 s
SMS-InI	5 mm × 5 mm × 5 mm	0.1 s

### SL and tSNR modulated by the regularization parameter

**Figure 4:**
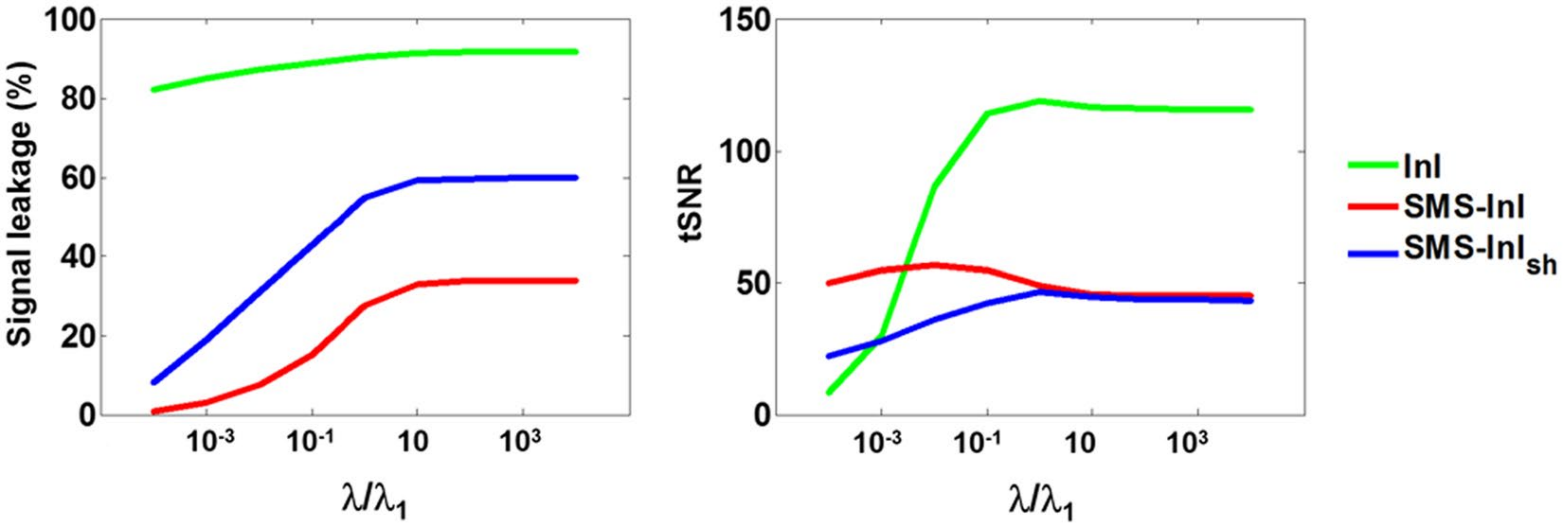
Time-domain SNR (*tSNR*) and average signal leakage (SL) varied as the regularization parameter changed for InI, SMS-InI, and single-shot SMS-InI (SMS-InI_sh_). Using the regularization parameter = 10^-2^ λ_1_ provided both high *tSNR* (57) and small spatial smearing (SL = 7%).

Figure 4 shows average *tSNR* and SL over the whole brain as a function of the chosen regularization parameter. Using the regularization parameter = 10^−4^ λ_1_ had *tSNR* = 50, which corresponded to a minor spatial smearing (average SL = 0.7%). The peak *tSNR* was 57 when images were reconstructed with the regularization parameter = 10^−2^ λ_1_, which yielded 7% average SL. Using the regularization parameter larger than 10^−2^ λ_1_ caused both unfavorable *tSNR* reduction and average SL growth. In short, we considered the regularization parameter = 10^−2^ λ_1_ as the optimal choice in terms of trading-off between SL and *tSNR* for SMS-InI.

**Figure 5:**
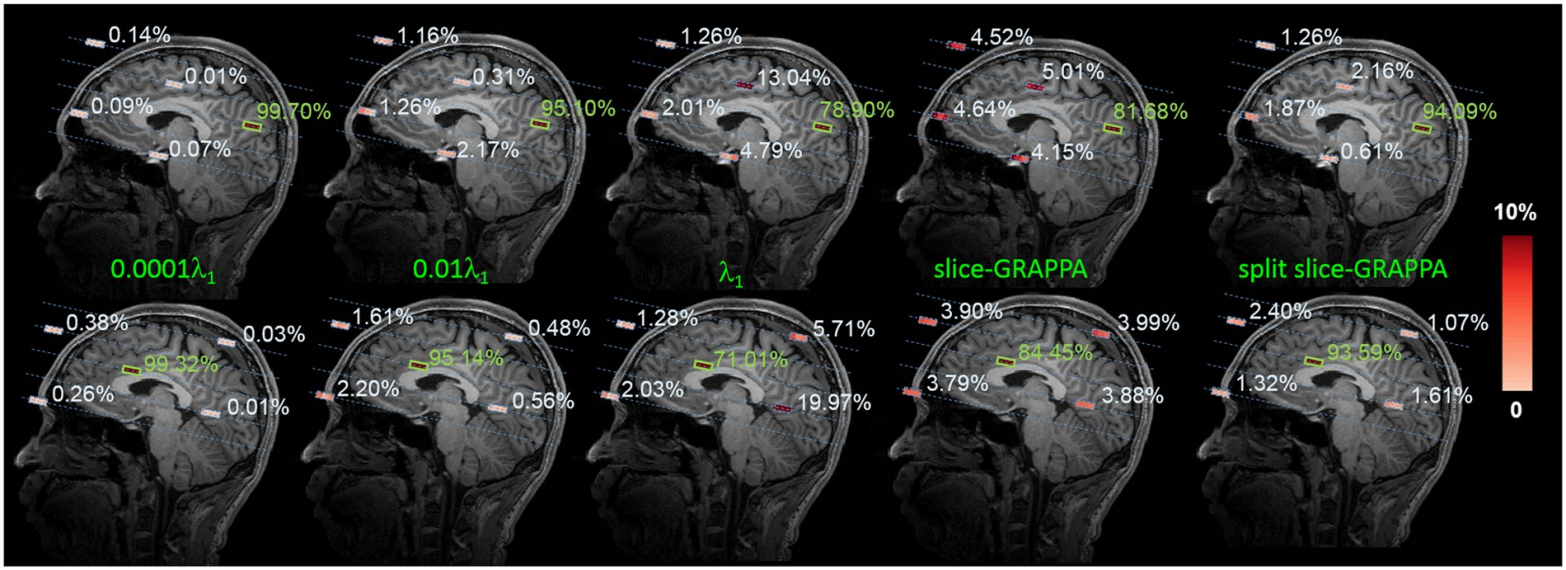
Spatial distributions of the signal leakage (SL) for SMS-InI reconstructed by the regularized SENSE method with different regularization parameters, by the slice-GRAPPA method, or by the split slice-GRAPPA method with the source at a cortical location (top row) or at a deep brain location (bottom row). The location of the true source was indicated by the thick green line, while four aliasing locations were indicated by the thick blue lines. These locations were distributed over 5 different slices indicated by blue dashed lines. The color codes the amplitude of the reconstructed signal. Ideally, the value at the source location is 1 and values at four other aliasing locations are 0. Low values in 4 aliasing locations suggest a small SL.

We calculated spatial distributions of the SL at two representative locations: one at the cortex and the other at the deep brain area. The SL corresponding to regularized SENSE reconstructions with λ = 10^−4^ λ_1_, 10^−2^ λ_1_, λ_1_, slice-GRAPPA reconstruction and split slice-GRAPPA reconstruction were depicted in Fig. 5. Note that the slice-GRAPPA reconstruction had much more serious signal leakage than regularized SENSE reconstructions, consistent with two recent studies^23,27^. Moreover, the split slice-GRAPPA reconstruction can reduce signal leakage about 10% compared with slice-GRAPPA reconstruction.

**Figure 6:**
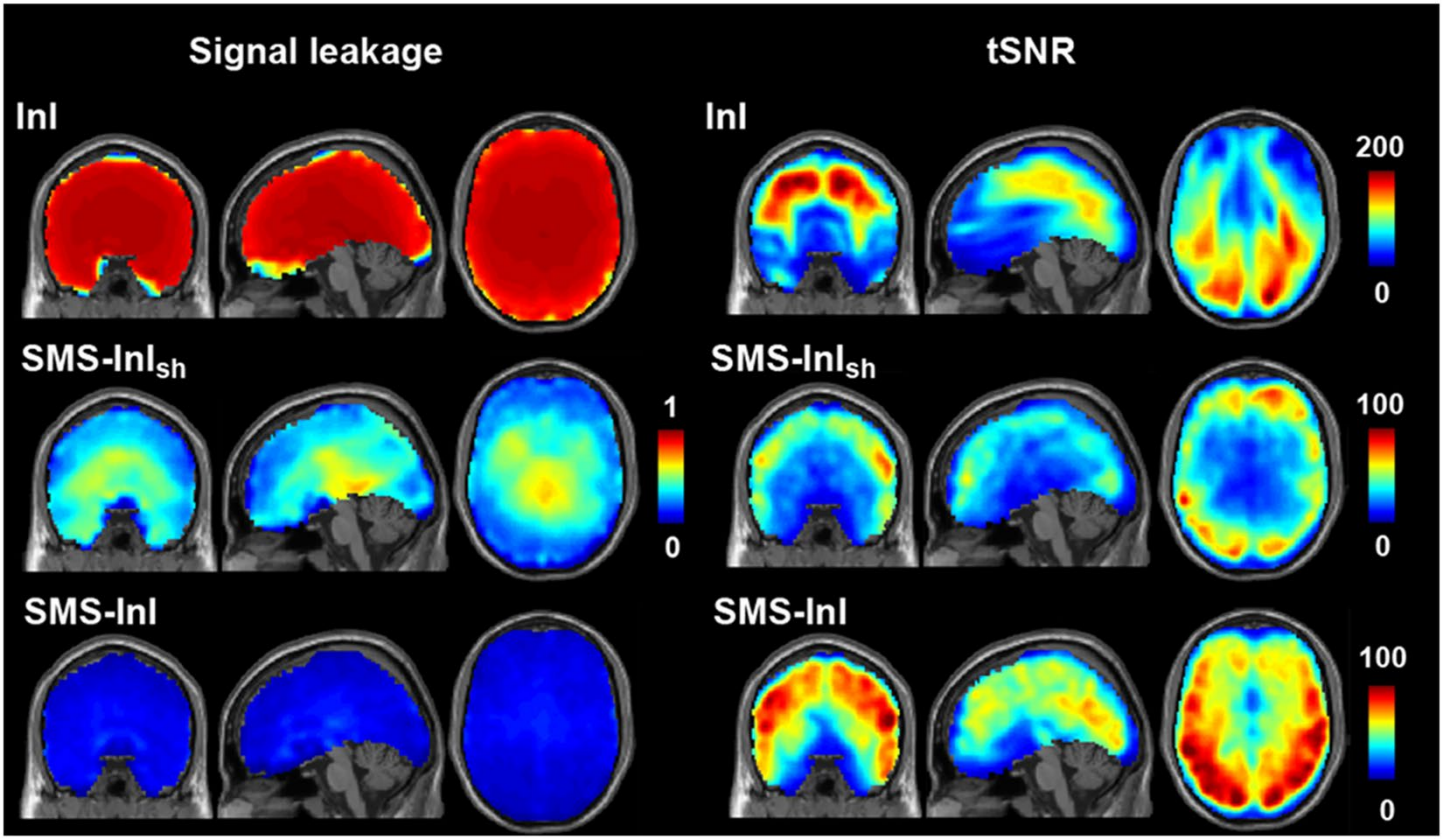
(Left) Maps of signal leakage for SMS-InI and InI with the optimized regularization parameter (10^−2^ λ_1_). (Right) Maps of *tSNR* for SMS-InI and InI with the optimized regularization parameter.

Figure 6 shows the spatial distribution of SL and *tSNR* for the optimal regularization parameter (10^−2^ λ_1_). The slice leakage is different for the set of image voxels that were aliased together. In fact, the pixel with higher coil sensitivity is less affected by regularization. As the image pixel around the center of the brain had low coil sensitivity, regularization affected stronger and caused larger signal leakage. The average and the standard deviation of the SL was 7% and 3%, respectively. The average and the standard deviation of the *tSNR* was 57 and 23, respectively. For comparison, the InI has SL of 87% + /− 11% and *tSNR* = 86 + /− 50. Importantly, the spatial distribution of SL in SMS-InI was much lower than in InI, suggesting that SMS-InI had a higher spatial resolution.

### fMRI Experiment 1

The purpose of this experiment was to test the spatial resolution of SMS-InI. Checkerboard flashing (8 Hz and 0.5 s duration) with either large (15.7°) or small (8.1°) visual angle was shown to the participants to elicit hemodynamic responses in the visual cortex. To keep visual fixation, participants were instructed to press a button when the color of the visual fixation crosshair changed. The same experimental paradigm was measured by SMS-InI, InI, and EPI, which was taken as the gold standard of spatial resolution. We hypothesized that i) acquisitions of accurate spatial resolution can reveal more extensive BOLD signal at the visual cortex by the large view angle stimuli, and ii) the BOLD signal distributions at both cortex and deep brain area should be similar to those revealed by EPI.

**Figure 7:**
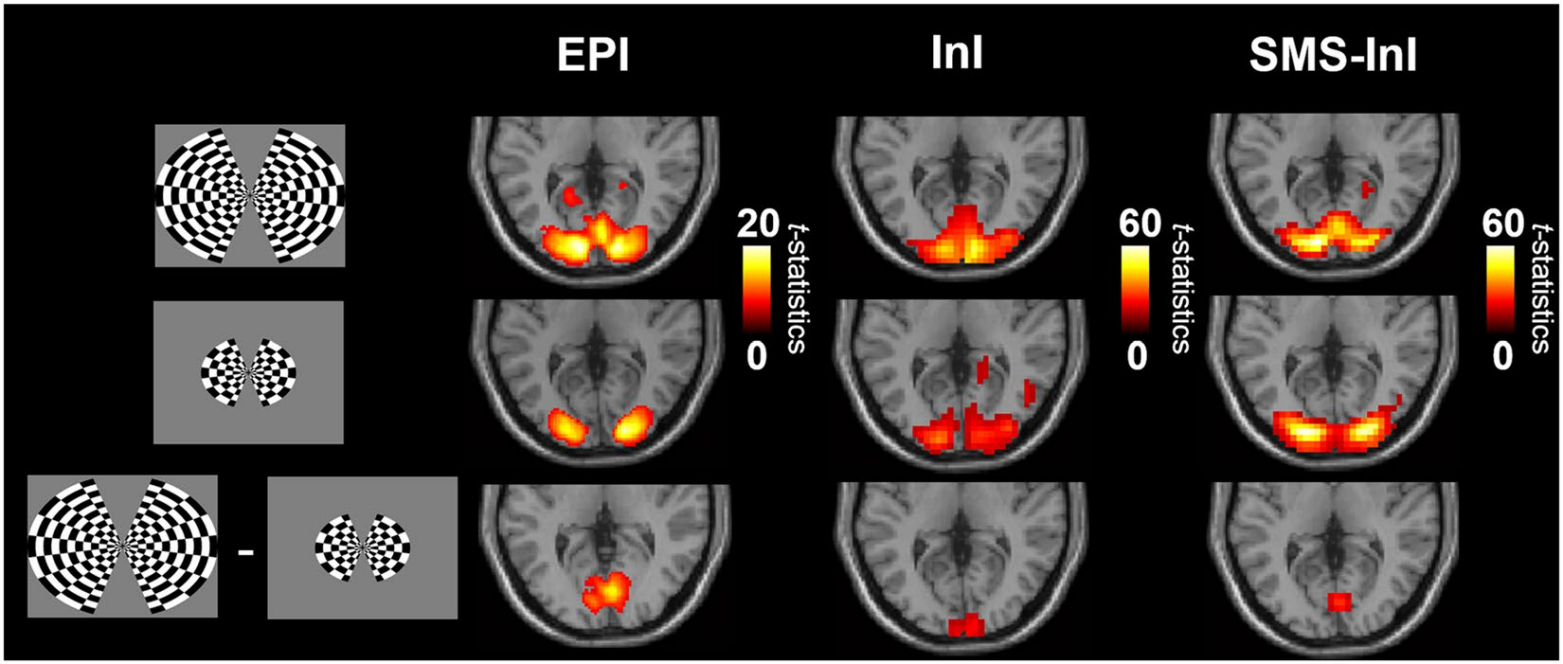
Significant BOLD signals measured by EPI, InI and SMS-InI elicited by visual stimuli with a large (15.7°; top row) or small (8.1°; middle row) view angle. Maps of statistically significant difference between these two stimuli conditions are shown at the bottom row.

Figure 7 shows the BOLD signal elicited by visual stimuli of two different sizes. Note that the color scale of t-statistics is different for EPI, InI, and SMS-InI. EPI, InI, and SMS-InI data all reported that the size of the functional activity at the visual cortex was modulated by the size of the stimuli. In particular, BOLD signal was found to extend more anteriorly along the central fissure when visual stimuli with a larger view angle were delivered. However, as we contrasted the BOLD signals elicited by stimuli of different view angles, we found that the spatial distribution of this significantly different BOLD signal was matched between EPI and SMS-InI data, while InI data showed a clear posterior shift. By taking the difference between two visual stimuli with large and small view angles, EPI localized brain areas that were selectively activated by the periphery of the checkerboard flashing pattern. As SMS-InI detected the matched brain areas, we had more confidence in the improved spatial resolution by SMS-InI.

**Figure 8:**
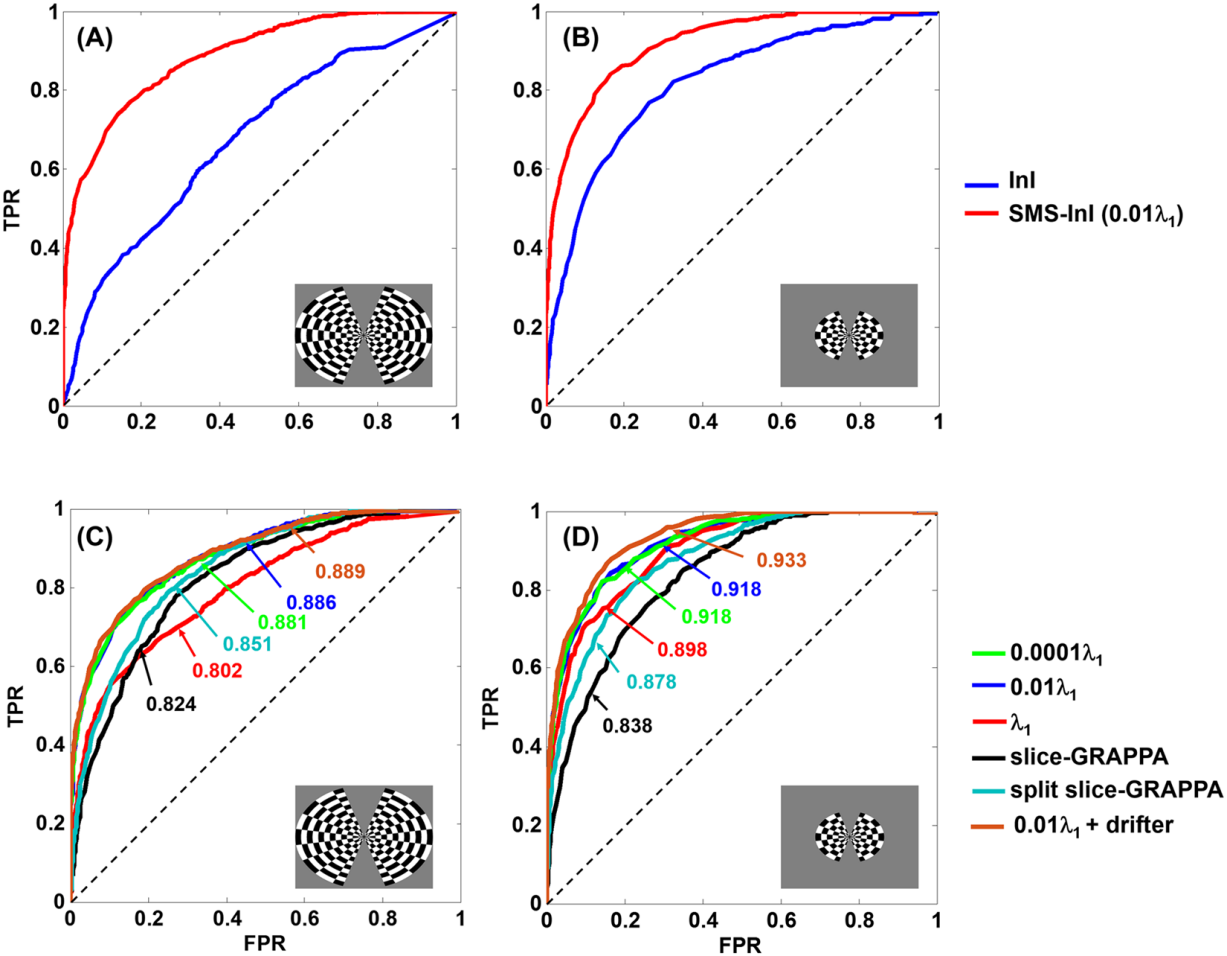
Receiver-operating-characteristic (ROC) curves between true-positive rate (TPR) and false-positive rate (FPR) for InI and SMS-InI measurements in detecting BOLD signals elicited by visual stimuli of large (**A**) or small (**B**) view angles. (**C**) and (**D**) are ROC curves for the SMS-InI data reconstructed by the regularized SENSE method with different regularization parameters and by the slice-GRAPPA and split slice-GRAPPA method for the large and small view angle stimuli, respectively.

To further quantify the detection power, we plotted ROC curves for InI and SMS-InI data with the visual cortex defined by EPI data as the gold standard. Figure 8A and B show that, with large view angle stimuli, the area under the ROC curve (AUC) for InI and SMS-InI were 0.67 and 0.89, respectively. With small view angle stimuli, the AUC for InI and SMS-InI were 0.82 and 0.92, respectively. These suggested that SMS-InI had a higher sensitivity and specificity in detecting BOLD signals at the visual cortex than InI.

Figure 8C and D depict the ROC curves for data reconstructed with different regularization parameters (10^−4^ λ_1_, 10^−2^ λ_1,_ and λ_1_) and reconstructions using the slice-GRAPPA method for experiments using large and small view angle stimuli. For the experiment using large view angle stimuli, the AUC for regularized SENSE with λ = 10^−4^ λ_1_, 10^−2^ λ_1,_ and λ_1_ were 0.881, 0.886, and 0.802, respectively. The largest AUC was found with λ = 10^−2^ λ_1_. After physiological noise cleaning, AUC was improve to 0.889. Reconstructions with the slice-GRAPPA and split slice-GRAPPA method had AUC = 0.824 and AUC = 0.851 respectively. Both are smaller than the AUC for regularized SENSE reconstruction with λ = 10^−2^ λ_1_. For the experiment using small view angle stimuli, the AUC for regularized SENSE with λ = 10^−4^ λ_1_, 10^−2^ λ_1,_ and λ_1_ were 0.918, 0.918, and 0.898, respectively. Both λ = 10^−4^ λ_1_ and λ = 10^−2^ λ_1_ gave the largest AUC, presumably because of the small SL such that the size of activated brain areas remained stable. Moreover, after physiological noise cleaning, the AUC was improved to 0.933. Reconstructions with the slice-GRAPPA and split slice-GRAPPA method had AUC = 0.838 and AUC = 0.878. Both are still smaller than the AUC for regularized SENSE reconstruction with λ = 10^−2^ λ_1_.

The sensitivity of reconstructions using the regularized SENSE reconstructions with different regularization parameters and using the slice-GRAPPA reconstructions were also compared by calculating the average *t*-statistics at the visual cortex ROI defined by the EPI (Table 2 **)**. This result also shows that regularized SENSE reconstructions are more sensitive in estimating the BOLD signal than slice-GRAPPA reconstructions in both experiments using either large or small view angle stimuli. Furthermore, data derived from reconstruction with the regularization parameter λ = 10^−2^ λ_1_ gave the higher *t*-statistics than reconstructions with the regularization parameter λ = 10^−4^ λ_1_ or λ = λ_1_, suggesting λ=10^−2^ λ_1_ can sensitively detect the BOLD response. Higher *t*-statistics is likely attributed to the increased *tSNR* by the regularization.

**Table 2:** The average *t*-statistics of detecting the visual cortex activity in response to small and large view angle stimuli using data reconstructed by the regularized SENSE method with different regularization parameters and by the slice-GRAPPA method.

Reconstruction method		Small view angle stimuli	Large view angle stimuli
Regularized SENSE	10^−4^ λ_1_	29.11	20.84
	10^−2^ λ_1_	30.16	21.06
	λ_1_	29.07	20.22
Slice-GRAPPA		17.28	12.59

The visual cortex region-of-interest was defined by the EPI data. λ_1_ is the largest eigenvalue of **A** ^*H*^ **C** ^−1^ **A**, where **A** is the image encoding matrix and **C** is the noise covariance matrix.

**Figure 9:**
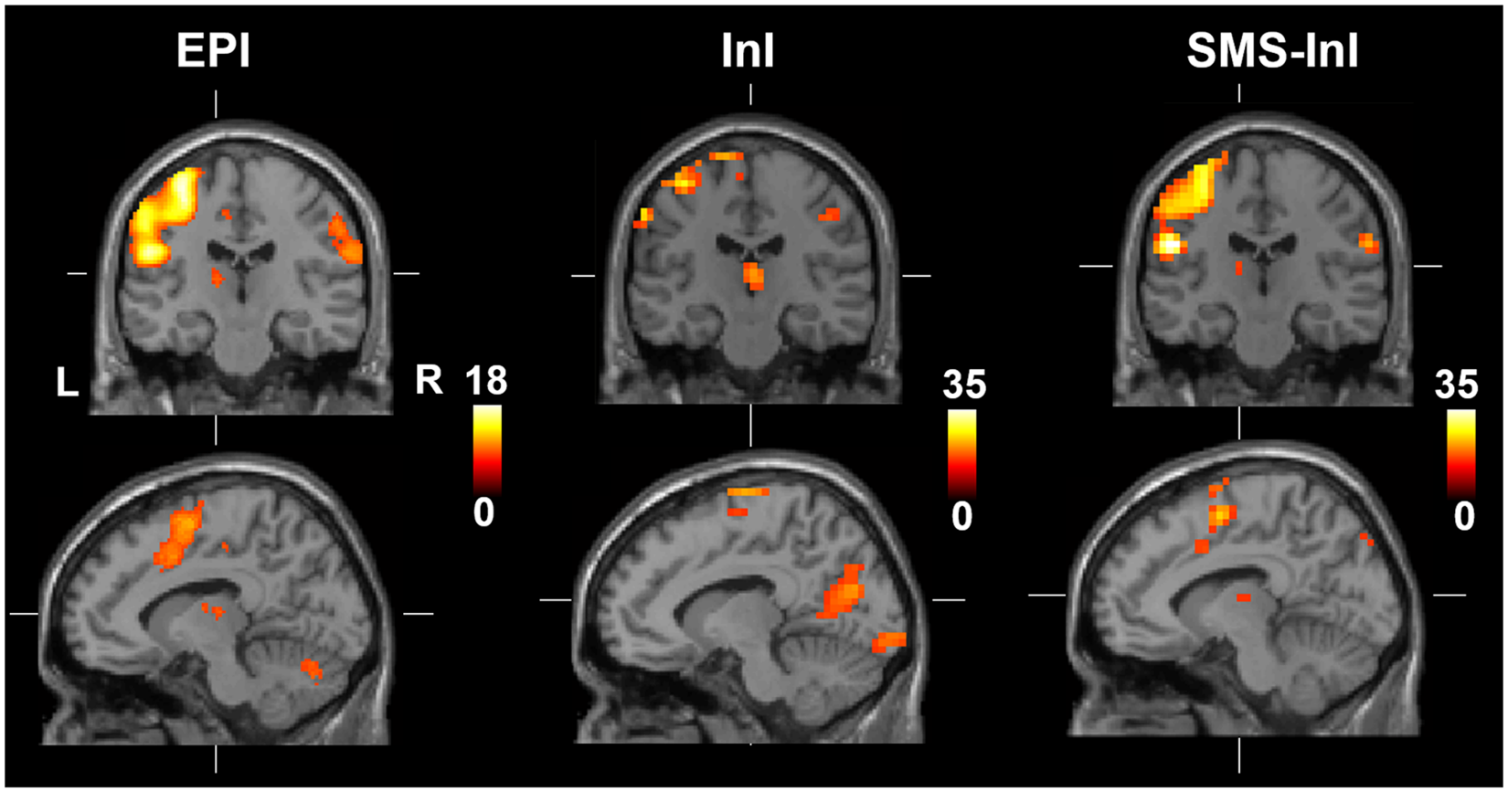
Significant BOLD signals were found in the sensorimotor cortices and thalamic areas by EPI and SMS-InI, while InI only detected significant BOLD signal at the sensorimotor area with a much smaller extend.

**Figure 10:**
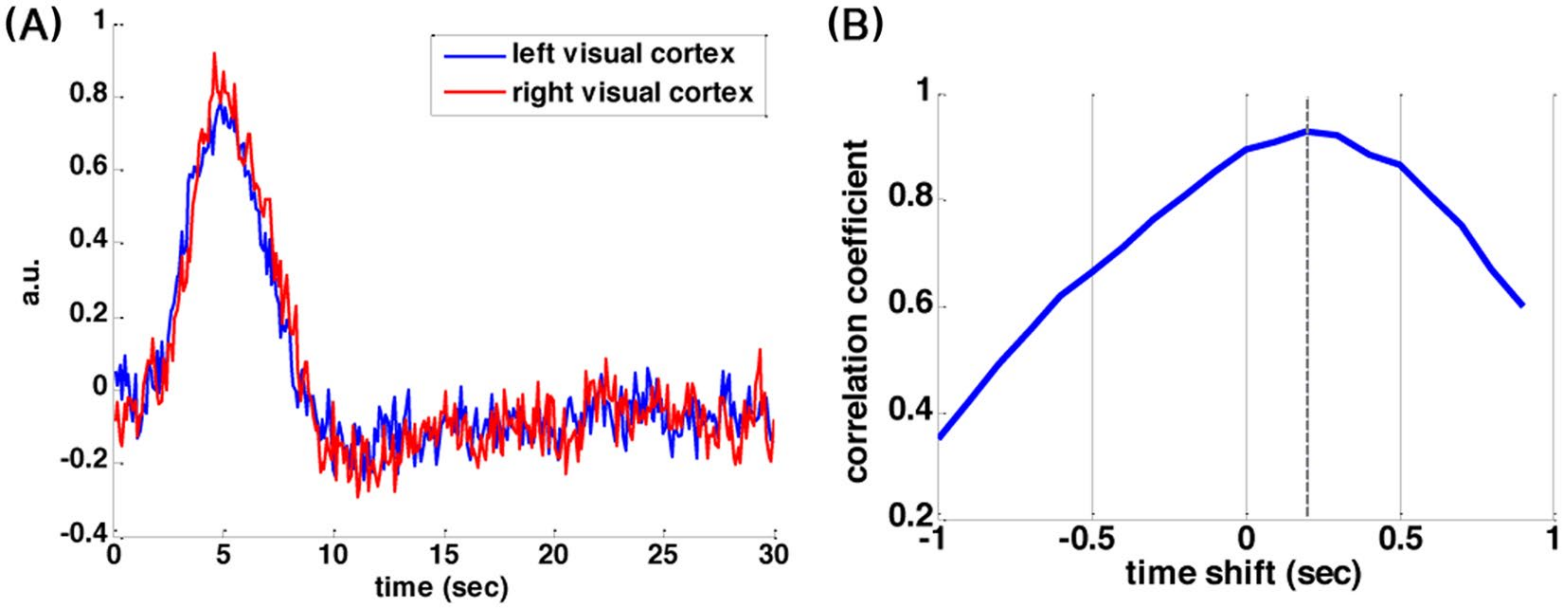
(**A**) BOLD signals in the left and right visual cortices elicited by hemi-field stimuli, where a 200 ms latency was between them. (**B**) These BOLD signals were found maximally correlated to each other when the hemodynamic response at the left visual cortex was delayed by 200 ms. Positive time shift indicates the hemodynamic response function at the left visual cortex preceded that at the right visual cortex.

We also studied the BOLD signal in the sensorimotor cortex using data collected in Experiment 1, where subjects performed a motor task to respond to color changes of the visual fixation crosshair. Figure 9 shows that the size of the significant BOLD signal at the sensorimotor cortex was found similar between EPI and SMS-InI data, while InI data estimated a much smaller area with significant BOLD signal in the motor cortex. Furthermore, Fig. 10 also shows that both EPI and SMS-InI detected thalamic BOLD signal at the same location. However, InI cannot reveal such subcortical activity.

### fMRI Experiment 2

The purpose of this experiment was to test the temporal resolution of SMS-InI. Two hemi-field visual checkerboard patterns separated by 0.2 s (right hemi-field stimulus followed by left hemi-field stimulus; 15.7° view angle; each hemifield stimulus 0.125 s duration) to the subjects. Using this design, we studied if the relative BOLD signal latency between left and right visual cortices represented the relative latency between left and right visual hemi-field stimuli.

Figure 10A shows the estimated hemodynamic response function (HRF) in left and right visual cortex. The two waveforms were visually similar but with a discernable latency between them. Detailed examination revealed that the HRF in the left visual cortex preceded that of the right visual cortex, matched to our experimental paradigm where right hemi-field stimuli were preceded to left hemi-field stimuli (by 200 ms). The cross-correlation analysis (Fig. 10B) revealed that these two waveforms were maximally correlated to each other when the hemodynamic response at the left visual cortex was delayed by 200 ms. Taken together, we found that the latency of the BOLD signal between bi-hemispheric visual cortices can follow the latency between the visual stimuli between two hemi-fields. And such a latency can be detected by SMS-InI.

## Discussion

This study proposed the SMS-InI method by optimizing the combination of SER, blipped-CAIPI-EPI, and highly parallel detection to achieve 100-ms whole-brain sampling of hemodynamic responses with 5-mm isotropic resolution. Our method had high *tSNR* (*tSNR* ~57; Fig. 4). In this study, we explicitly investigated the trade-off between *tSNR* and signal leakage associated with the regularization parameter in blipped-CAIPI reconstruction (Fig. 4). In particular, high *tSNR* comes at the price of large signal leakage and small signal leakage comes at the price of low *tSNR*. This trade-off is important in fMRI experiments concerning the significance of BOLD signal estimates and spatial resolution. SMS-InI reconstructed by the regularized SENSE method was compared with the slice-GRAPPA method^18^. Results showed that the regularized SENSE reconstructions had lower signal leakage (Figs 2 and 3), more sensitive detection of the BOLD signal (Table 2), and higher detection power (Fig. 8C and D) than data reconstructed by the slice-GRAPPA method. Empirical results of detecting significant brain responses at cortical (Figs 7 and 9) and subcortical areas (Fig. 9) with comparable results measured by EPI suggested that the spatial resolution of SMS-InI can be applied to versatile experiments concerning brain activity across the whole brain. The high sampling rate of SMS-InI allowed us to reveal that the hemodynamic responses at left and right visual cortices had a relative 200-ms latency matched to the stimuli latency at right and left hemi-fields (Fig. 10). Taken together, we consider that SMS-InI is an improved fast fMRI acquisition with whole-brain sampling in the range about 100 ms.

The optimized 2-shot SMS-InI consisted of two acquisition modules, each of which took 50 ms and imaged ten 5-mm thick slices, to achieve whole-brain coverage and a 10 Hz sampling rate. SMS-InI can also achieve single-shot imaging of the same spatiotemporal resolution by the combination of SER, blipped CAIPI-EPI, and the optimized regularization parameter. Such a strategy relies more on the coil sensitivity information to separate between aliased slices than the 2-shot optimized SMS-InI. Consequently, the single-shot SMS-InI (SMS-InI_-_sh) had lower *tSNR* and larger SL than optimized 2-shot SMS-InI. Figure 4 also shows how *tSNR* and SL were modulated by different choices of the regularization parameter in single-shot SMS-InI. Quantitatively, the *tSNR* and SL for single-shot SMS-InI using regularization parameter 10^−2^ λ_1_ were 36 and 31%, respectively. They had lower *tSNR* and larger SL than 2-shot optimized SMS-InI.

We were aware of a previous study using combined SER and blipped CAIPI EPI to sample BOLD signal in intervals of 100 ms with whole-brain coverage^7^, similar to the single-shot SMS-InI discussed above. They found that the *tSNR* was only about 20. This is likely because of the higher image resolution (2.5 mm × 2.5 mm × 3 mm) used in their study. In this study, single-shot SMS-InI with the optimized regularization with 5 mm × 5 mm × 5 mm resolution had *tSNR* = 30. However, caution must be made in comparison between these two methods, because regularization parameter plays an important role in both *tSNR* and spatial smearing.

Previously, using 6-channel coil array for 2-fold accelerated cardiac imaging on 1.5 T, it was found that 5% of the first eigenvalue of **A** ^*H*^ **C** ^−1^ **A** was a good choice of the regularization parameter^24^. In this study, the optimal regularization parameter was about 1% of the first eigenvalue of **A** ^*H*^ **C** ^−1^ **A**: this regularization parameter resulted in the highest *tSNR* with minor SL (Fig. 4). Reconstructed images with this regularization parameter also had the largest *t*-statistics (Table 2) and the highest power in detecting the BOLD response (Fig. 8C and D). The optimal regularization was consistent between our study and experiments reported by Sodickson *et al*., considering the difference of coil array, acceleration rate, and field strength.

The close relationship between SMS-InI and our previous InI methods is the regularized reconstruction of multi-channel coil array measurements. While 2-shot SMS-InI can achieve a high *tSNR* and small SL by a relatively minor regularization (10^−2^ λ_1_), a much stronger regularization (λ_1_) is needed for single-shot SMS-InI and both InI to achieve the highest *tSNR* (Fig. 4). With different choices of spatial resolution (in-plane resolution, slice thickness, and gap), we expect that the regularization parameter should be optimized differently.

The novelty of SMS-InI is its high spatiotemporal resolution (10 Hz whole-brain coverage sampling rate with nearly isotropic 5-mm spatial resolution) enabled by the optimally regularized parallel imaging reconstruction with high computational efficiency. While blipped CAIPI-EPI has been used extensively nowadays, its sampling interval was typically around 300 ms or longer with about 3 mm spatial resolution. For fMRI experiments interested in fine temporal features in the hemodynamics afforded by acquisitions with the repetition time (TR) of 0.1 s or shorter, different methods, such as echo-volumar imaging (EVI)^28^, MR-encephalography (MREG)^13^, and inverse imaging (InI)^14^ have been attempted. Yet none of these methods can achieve the spatiotemporal resolution reported in this study. Specifically, InI suffers from anisotropic resolution (5 mm at cortex but about 20 mm at subcortical areas). MREG has the challenges of image blurring and long image reconstruction time (hours for one brain volume). EVI has different blurring/distortion artifacts along encoding directions. SMS has been tuned to achieve TR = 0.1 s. However, its *tSNR* was too low to generate useful fMRI data and the associated spatial resolution was not quantitatively characterized^7^. Here SMS-InI carefully combines SMS, SER, and regularized reconstruction to address these issues. The SMS-InI reconstruction can be separated into many independent sets of aliased image voxels related by an encoding matrix of small dimension (32 × 5 in this study). Because of this separation and the analytical solution (Eq. 1), reconstructing a volume of SMS-InI took less than 0.1 seconds. The performance of SMS-InI was quantitatively assessed using *tSNR* and the point spread function (quantified by the “signal leakage” index) with various regularization parameters (Fig. 4). The optimally regularized SENSE reconstruction of SMS-InI provided small signal leakage (Fig. 5), sensitive (Table 2) and powerful detection of the BOLD responses (Fig. 8C and D). These results outperformed the reconstructions using the slice-GRAPPA method^18^ and the split slice-GRAPPA method^23^ as the signal leakage was minimized. Experimental data were provided to demonstrate that high spatiotemporal resolution was achieved at both cortical and subcortical regions. Additionally, we presented two versions of SMS-InI: the 2-shot SMS-InI offered nearly isotropic 5-mm spatial resolution, while the single-shot SMS-InI was capable of 20 Hz sampling rate at the cost of reduced spatial resolution and *tSNR*. Taken together, we attempted to introduce SMS-InI as a novel fMRI acquisition tool to provide high spatiotemporal resolution measurements of hemodynamics.

We have attempted to improve the spatial resolution of InI through different image reconstruction algorithms^14,29,30^. However, these efforts still suffer from the significant spatial resolution loss at deep brain areas. To address this challenge, we have also tried i) combining acquisitions of different “projection” directions^31^, and ii) using blipped-CAIPI in accelerated 3D echo-volumnar imaging readouts^7^. The former approach indeed improved spatial resolution of InI. However, at deep brain areas, the effective spatial resolution was still as coarse as 16 mm after combining three orthogonal projections. The latter approach reported point spread functions of about 10 mm and 16 mm for whole-brain and subcortical areas, respectively. The multiple-projection InI method^31^ needs to acquire data from independent runs and thus motion artifacts and system stability are both practical concerns to ensure the consistency required in image reconstruction.

The blipped-CAIPI InI^7^ reported previously may seem similar to SMS-InI. Yet there are a few major differences that we would like to emphasize. First, there was no SER in our blipped–CAIPI InI. Since SER can separate data from neighboring slices *without* using coil sensitivity, SMS-InI can more effectively separate next-to-neighboring slices using coil sensitivity. Second, the gradient moments at the partition/slice direction in SMS-InI were similar to those in blipped-CAIPI InI. Thus, for example, two slices separated by 20 mm had similar 1/3 FOV shift in both SMS-InI and blipped-CAIPI InI. However, blipped CAIPI-InI received signals from as many as 5 slices across 20 mm. Intermediate slice images (slices 2, 3, and 4) were spatially modulated by a point spread function as a complex weighted combination of a discrete delta function without shift and a discrete delta function with 1/3 FOV shift. On the contrary, SMS-InI had no signal from intermediate slices because of 2-shot slice group prescription and SER. This explains why SMS-InI has much improved spatial resolution than blipped-CAIPI InI.

Our study suggested SMS-InI parameters to have 5-mm isotropic resolution with minimal point-spread function. Such a spatial resolution is not high compared with fMRI studies with sub-millimeter resolution^32^. However, among cognitive neuroscience experiments, acquisitions with slice thickness of about 3-4 mm (including gaps) together with whole-brain coverage using 64 × 64 image matrix were still quite common^33^. Further considering typical spatial smoothing used in the post-processing, the eventual spatial resolution was typically no higher than 5 mm. Therefore, we consider our SMS-InI offers a reasonable spatial resolution to fMRI experiments.

The comparison between SMS-InI and standard 2D EPI (Figs 7 and 9) was meant to use the standard 2D EPI as the relatively high spatial resolution measurements to demonstrate that the functional brain areas characterized by SMS-InI were similar to those in 2D EPI. On the other hand, the comparison between SMS-InI and InI (Figs 7, 8, and 9) was meant to demonstrate how the spatial resolution was improved in SMS-InI with the same sampling rate in both acquisitions (10 Hz). In other words, SMS-InI can detect similar cortical and subcortical BOLD signals like EPI and has a similar temporal resolution like InI.

In this study, we used the regularized SENSE reconstruction, which did not correct artifacts related to eddy currents and other sources of off-resonance. Recently, hybrid SENSE^20,34^ was proposed to reduce image distortion. In the future, we may replace slice-GRAPPA/split slice-GRAPPA with hybrid SENSE to further improve the geometric fidelity of the reconstructed images.

Opportunities for further improving the temporal resolution are: i) one-shot SMS-InI, where the readout can be as short as 50 ms per volume. However, the single-shot SMS-InI had lower *tSNR* and larger SL than 2-shot SMS-InI. ii) incorporating echo-shifting^35^ similar to what we have tried previously^15,36^. We expect that echo-shifting can achieve TR = 20 ms and TE = 35 ms at the cost of reduced *tSNR*. Note that both possible developments will try to achieve the optimal TE at 3 T (~30 ms).

Spatial resolution may be improved by i) using a coil array of similar coverage and size but more channels in order to improve the spatial encoding by more localized coil sensitivity, ii) using SMS-InI at higher field because the coil sensitivity becomes more disparate among channels with a shorter wavelength. Both strategies aim at improving the conditioning of the encoding matrices. The spatial resolution can also be improved by using a longer readout, which needs to tolerate a longer TE and a larger echo time difference between slices separated by SER. Using a gradient coil with a stronger slew rate and data sampling during the ramping-up phase of the gradient may partially mitigate these two challenges.

In conclusion, SMS-InI is a method combining simultaneous multi-slice, blipped-CAIPI EPI, and regularized image reconstruction. With whole-brain coverage, 5-mm isotropic spatial resolution, and 10 Hz sampling, SMS-InI can be a useful tool in detecting cortical and subcortical hemodynamic responses with high spatiotemporal resolution.
